# HIV-1 Disease Progression and Drug Resistance Mutations among Children on First-Line Antiretroviral Therapy in Ethiopia

**DOI:** 10.3390/biomedicines11082293

**Published:** 2023-08-18

**Authors:** Yimam Getaneh, Fentabil Getnet, Feng Ning, Abdur Rashid, Lingjie Liao, Feng Yi, Yiming Shao

**Affiliations:** 1 State Key Laboratory for Diagnosis and Treatment of Infectious Diseases, National Clinical Research Center for Infectious Diseases, Collaborative Innovation Center for Diagnosis and Treatment of Infectious Diseases, The First Affiliated Hospital, College of Medicine, Zhejiang University, Hangzhou 310027, China; 12018626@zju.edu.cn; 2 Ethiopian Public Health Institute, Addis Ababa P.O. Box 1242, Ethiopia; 3 Takemi Program in International Health, Harvard T.H. Chan School of Public Health, Boston, MA 02115, USA; 4 Chinese Center for Disease Control and Prevention, Beijing 102206, China; 5 School of Medicine, Nankai University, Tianjin 300071, China; 6 State Key Laboratory for Infectious Disease Prevention and Control, National Center for AIDS/STD Control and Prevention, Chinese Center for Disease Control and Prevention, Beijing 102206, China

**Keywords:** HIV/AIDS, children, disease progression, drug resistance, Ethiopia

## Abstract

**Background:** High rates of disease progression and HIV drug resistance (HIVDR) among adults taking highly active antiretroviral treatment (HAART) in Sub-Saharan Africa were previously documented. However, children were generally not considered despite their greater risk. Hence, this study was aimed to evaluate HIV-1 disease progression and drug resistance mutation among children on first-line antiretroviral therapy in Ethiopia. **Method:** A longitudinal study was conducted among 551 HIV-positive children (<15 years old) recruited between 2017 and 2019 at 40 antiretroviral treatment delivery sites in Ethiopia. Disease progression was retrospectively measured over a 12-year (2007–2019) follow-up as the progress towards immunosuppression. Two consecutive viral load (VL) tests were conducted in 6-month intervals to assess virologic failure (VF). For children with VF, HIV-1 genotyping and sequencing was performed for the *pol* gene region using in-house assay validated at the Chinese Center for Disease Control and Prevention, and the Stanford HIVDB v9.0 algorithm was used for identification of drug resistance mutations. The Kaplan–Meier analysis and Cox proportional hazards regression model were used to estimate the rate and predictors of disease progression, respectively. **Results:** The disease progression rate was 6.3 per 100 person-years-observation (95% CI = 4.21–8.53). Overall immunosuppression (CD4 count < 200 cells/mm^3^) during the 12-year follow-up was 11.3% (95% CI = 7.5–15.1). Immunosuppression was significantly increased as of the mean duration of 10.5 (95% CI = 10.1–10.8) years (38.2%) to 67.8% at 12 years (*p* < 0.001). Overall, 14.5% had resistance to at least one drug, and 6.2% had multi-drug resistance. A resistance of 67.8% was observed among children with VF. Resistance to non-nucleotide reverse transcriptase inhibitors (NNRTI) and nucleotide reverse transcriptase inhibitors (NRTI) drugs were 11.4% and 10.1%, respectively. Mutations responsible for NRTI resistance were M184V (30.1%), K65R (12.1%), and D67N (5.6%). Moreover, NNRTI-associated mutations were K103N (14.8%), Y181C (11.8%), and G190A (7.7%). Children who had a history of opportunistic infection [AHR (95% CI) = 3.4 (1.8–6.2)], vitamin D < 20 ng/mL [AHR (95% CI) = 4.5 (2.1–9.9)], drug resistance [AHR (95% CI) = 2.2 (1.4–3.6)], and VF [AHR (95% CI) = 2.82 (1.21, 3.53)] had a higher hazard of disease progression; whereas, being orphan [AOR (95% CI) = 1.8 (1.2–3.1)], history of drug substitution [(AOR (95% CI) = 4.8 (2.1–6.5), hemoglobin < 12 mg/dL [AOR (95% CI) = 1.2 (1.1–2.1)] had higher odds of developing drug resistance. **Conclusions:** Immunosuppression was increasing over time and drug resistance was also substantially high. Enhancing routine monitoring of viral load and HIVDR and providing a vitamin-D supplement during clinical management could help improve the immunologic outcome. Limiting HAART substitution is also crucial for children taking HAART in Ethiopia.

## 1. Background

Despite the remarkable achievements to halt the human immunodeficiency virus (HIV) epidemic globally, Sub-Saharan Africa (SSA) is still the most disproportionately affected region, where more than 72% of the global people living with HIV (PLHIV) reside [1,2,3]. Ethiopia is one of the highly affected countries on the continent, with an estimated prevalence of 0.96% (0.62% in rural and 3.0% in urban) in 2022, equivalent to 609,349 PLHIV [3,4].

Highly active antiretroviral therapy (HAART) remains the backbone for prevention of HIV progression to acquired immunodeficiency syndrome (AIDS) [5]. HAART helps to boost the immune system, which results in viral load suppression and CD4 cell count increment [6]. Viral load and CD4 cell counts are key prognostic markers for measuring HIV disease progression and HAART response [7,8]. The World Health Organization (WHO) recommends the use of both CD4 and viral load for monitoring HIV disease progression [9], but CD4 cell counts have been conventionally used as the main marker [5].

Despite a rapid scale-up of HAART in Ethiopia over the past years [6], its coverage has never been optimal. The estimated HAART coverage was 84% for adults and only 23% for children in 2018 [6]. Furthermore, the growing emergence of HIV drug resistance (HIVDR) has been a major challenge to the national HAART program [10,11,12,13,14]. This could be due to the use of drugs with low genetic barriers such as non-nucleotide reverse transcriptase inhibitors (NNRTI) [15]. The use of nevirapine as monotherapy for preventing mother-to-child transmission may also contributed to higher emergence of drug resistance in pediatric HIV [16]. Viral replication under sub-optimal antiretroviral pressure leads to accumulation of resistance mutations, which limit future therapeutic choices [17], as mutations conferring resistance to one drug frequently confer cross-resistance to other drugs within the same classes of antivirals [18]. Hence, HIV treatment outcomes, measured in terms of viral suppression and immunologic outcomes, would be lower in children than adults.

However, there is limited empirical evidence on immunologic outcome and drug resistance among children in Ethiopia. Therefore, this study aimed to describe HIV-1 disease progression and drug resistance and evaluate the effect of drug resistance on disease progression among children on first-line antiretroviral therapy in Ethiopia.

## 2. Methods

### 2.1. Study Setting

The Ethiopian government began a limited program of making HAART available on a fee basis in 2003, and in early 2005, began to make ART available on a free basis nationwide, with support from the Global Fund to Fight AIDS, tuberculosis and malaria [19]. As of June 2020, a total of 464,762 PLHIV were taking HAART [5,9]. According to the latest revised guideline [4], there were four allowable first-line ART regimens added for selective use in situations where NRTI-based regimens cannot be used or cause adverse effects or intolerance: d4T + 3TC + NVP, d4T + 3TC + EFV, ZDV + 3TC + EFV, and ZDV + 3TC + NVP. In the event of failure of the first-line regimen, the allowable second-line regimens are: (ddI or TDF) + ABC + (LPV/r or SQV/r or NFV or IND/r [4].

### 2.2. Study Design

A longitudinal study was conducted from 2017 to 2019 among HIV-infected children (<15 years old) on first-line antiretroviral therapy (ART). It was part of a national cohort study on HIV-1 treatment failure and acquired drug resistance among people taking ART at 63 health facilities in the country [19].

### 2.3. Study Population and Sampling

**Figure 1:**
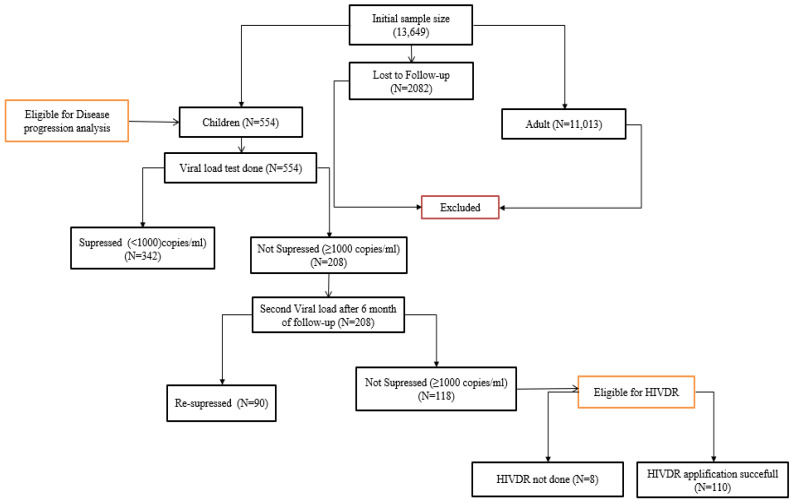
Eligibility, sampling, and sample size determination for disease progression and HIV drug resistance among children taking HAART in Ethiopia (2007–2019).

HIV-infected children taking first-line ART for at least six months were included in the study. Out of the 63 health facilities and 13,649 patients in the initial study, 40 had pediatric ART service and all the children from these health facilities (*n* = 554) were included. The disease progression of HIV-infected children was retrospectively retrieved for 12 years (2007–2019) and measured as a change in immunosuppression (i.e., CD4 count). CD4 count <200 cells/mm^3^ was used to define immunosuppression [20]. Moreover, viral load (VL) testing was conducted for children twice at 6-month intervals between March and September 2019 to determine virologic failure (VF). Then, children with VF (i.e., viral load ≥ 1000 copies/mL) were included for HIV drug resistance investigation, given those with VL < 1000 copies/mL were considered as susceptible and excluded from HIVDR testing [20] (Figure 1).

### 2.4. Data Collection

Data were collected using interview, medical record review, and laboratory testing. Mothers/female guardians of the children were interviewed during the routine follow-up visit to collect data on sociodemographic and individual factors. Patient records were also abstracted to capture data on clinical and laboratory results such as hemoglobin, CD4 count, and history of ART regimen. Then, children with initial viral load ≥1000 copies/mL at baseline testing were further followed for six months for a second round of VL re-testing. Enhanced adherence and counseling were introduced as interventions during the six-month follow up.

### 2.5. Laboratory Testing

Whole blood was collected from children using two ethylenediaminetetraacetic acid (EDTA) coated test tubes of 3–5 mL each, and plasma was extracted using centrifugation at 2000 revolutions per minute (RPM). The second-round blood samples were collected after a 6-month follow-up from children with an initial viral load >1000 copies/mL.

HIV-1 viral load tests at baseline and after 6-month follow-up were performed at selected regional laboratories in the country using two techniques (i.e., COBAS^®^ AmpliPrep/CobasTaqman^®^ and Abbott Real Time HIV-1). CD4 T-cell count and hemoglobin tests were conducted from whole blood at the study facilities using a Facscount^®^ automated cell counter (Becton-Dickinson, Franklin Lakes, NJ, USA) and CELDYN^®^ hematology analyzer, respectively. Moreover, inflammatory biomarker (hsCRP) and vitamin-D tests were performed at the national Clinical Chemistry laboratory using an Elecsys 2010 Clinical Chemistry Analyzer (Roche, Basel, Switzerland).

In-house assay validated by the Chinese Center for Disease Prevention and Control (CCDC) was used for amplification and sequencing of the *pol* gene of HIV-1 using an ABI-3730 DNA genetic analyzer [3]. The *pol* gene region of the HIV-1 gene was sequenced by an ABI-3730 DNA genetic analyzer [21,22]. The sequence was edited using a web-based ReCall (http://pssm.cfenet.ubc.ca/, accessed on 11 April 2023).

### 2.6. Variables

We had two outcome (dependent) variables; disease progression (i.e., CD4 count) and drug resistance. Independent variables were demographic characteristics (age, gender, education, and region); individual factors (previous ART exposure, missed appointments, and HIV status disclosure); and clinical characteristics (functional status, hemoglobin, clinical stage, and ART regimen history).

### 2.7. Definition

**Immunosuppression**: CD4 count of <200 cells/mm^3^ after 6 months of HAART initiation [23].

**Disease progression rate**: Overtime change in immunosuppression (CD4 count < 200 cells/mm^3^) of children [10].

**Virologic Failure**: Children with two consecutive plasma viral loads ≥ 1000 copies/mL within 6 months of follow-up with adherence and counseling [23].

**Drug resistance:** characterized according to the Stanford database (HIVDB). Since HIVDR testing was conducted for children with VL ≥ 1000 copies/mL, children with VL < 1000 copies were considered to be susceptible and included in the overall HIVDR analysis [23].

**Vitamin-D deficiency**: A plasma vitamin-D level of below 20 ng/dL [10,18].

**Inflammation**: A plasma hsCRP value of >3 mg/dL [10,18].

**Functional status**: ‘ambulatory’ if a patient was bedridden for <15 days of the month prior to recruitment to the study and ‘bedridden’ if the patient was bedridden for ≥15 days of the month prior to the study, as reported by the physician at the ART site [24].

### 2.8. Statistical Analysis

Data were summarized using median and mean for continuous and frequency and percentage for the categorical variables. The Kaplan–Meier curve was carried out to illustrate the rate of the disease progression among children, which was calculated as the rate of children with a CD4 count <200 cells/mm^3^ on a yearly basis, and the Cox proportional hazards regression model was used to determine the predictors of disease progression. Adjusted hazard ratios (HRs) with 95% confidence intervals were used to report effect sizes at a statistical significance level of *p* < 0.05.

Drug resistance was calculated using the HIVDB algorithm (v9.0) at Stanford’s HIV genotypic resistance profile (https://hivdb.stanford.edu/, accessed on 11 April 2023). The logistic regression model was used to identify factors contributing to drug resistance. Variables with *p* < 0.2 in crude analysis were included in the multivariable model and adjusted odds ratios (AOR) with 95% CIs were used to report effect sizes at a significance level of *p* < 0.05. All data analysis was conducted using STATA version 16.0.

## 3. Results

### 3.1. Demographic and Clinical Characteristics

The mean age of children was 9.33 (SD ± 2.21) years old. More than half of the study participants were aged below 10 years (58.3%), while the majority were urban dwellers (89.7%). Of all children on HAART, nearly half (46.8%) were from three regional states, Oromia (18.1%), Amhara (16.7%), and Southern Nations Nationalities and Peoples Region (SNNPR) (12%) (Supplemental Table S1).

A majority of the children (80%) were taking one of the three HAARTs regimens, which were Zidovudine (AZT) + Lamivudine(3TC) +Nevirapine (NVP) (35.9%), Stavudine(D4T) + Lamivudine(3TC) + Nevirapine (NVP) (30.3%), and Zidovudine (AZT) + Lamivudine(3TC) + Efavirenz (EFV) (14.0%) (Table 1; Supplemental Table S1). About half (47.5%) of the children had at least a one-time HAART substitution history. Overall, 63% of the substitutions were from those who were taking Abacavir (ABC) + Lamivudine(3TC) + Nevirapine (NVP) followed by Abacavir (ABC) + Lamivudine(3TC) + Efavirenz (EFV) (21.2%), and Tenofovir (TDF) + Lamivudine(3TC) + Nevirapine (NVP) (16.0%). Moreover, more than half of 56% of the substitutions were replaced by Stavudine(D4T) + Lamivudine(3TC) + Efavirenz (EFV) followed by Stavudine(D4T) + Lamivudine(3TC) + Nevirapine (NVP) (31.31%) and Zidovudine (AZT) + Lamivudine(3TC) + Nevirapine (NVP) (12%). At baseline of the study, 37.8% of the children had a viral load >1000 copies/mL, which was reduced to 21.4% after 6 months of follow-up, when adherence and counseling interventions were provided. More than half (61.3%) of the children had inflammation (hsCRP > 3.0 mg/L) and 51.5% had vitamin-D deficiency (Vitamin D < 20 mg/L) (Table 1).

### 3.2. Disease Progression

Overall immunosuppression among children taking HAART in Ethiopia was 11.25% (95% CI = 7.5–15.1). Among male children, immunosuppression was 12.95% compared to 9.52% among female. Immunosuppression was also higher in the age group 6–10 years (12.24%) compared to ≤5 years (6.58%). Children with a history of opportunistic infections (OIs) had a 22.64% level of immunosuppression compared to 8.54% among those who had no OIs. Of those children who were not virally suppressed, 26.27% had immunosuppression. Children with hsCRP >3 mg/L and those with vitamin D <20 ng/mL had a higher rate of immunosuppression, which accounted for 338 (13.31%) and 284 (18.66%), respectively. A quarter of the children with a duration on HAART for more than 133 months had immunosuppression (Supplemental Table S2).

**Figure 2:**
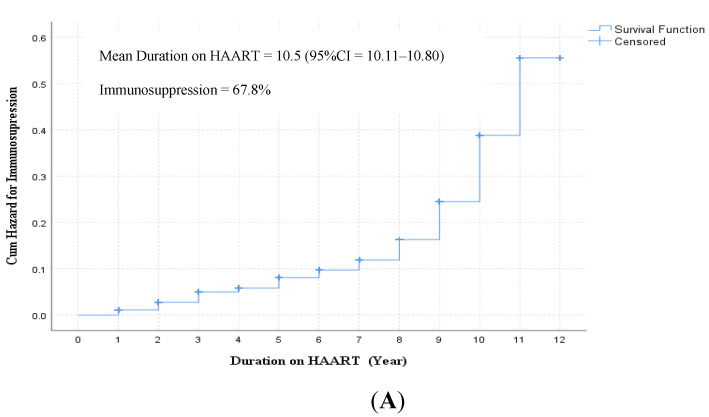

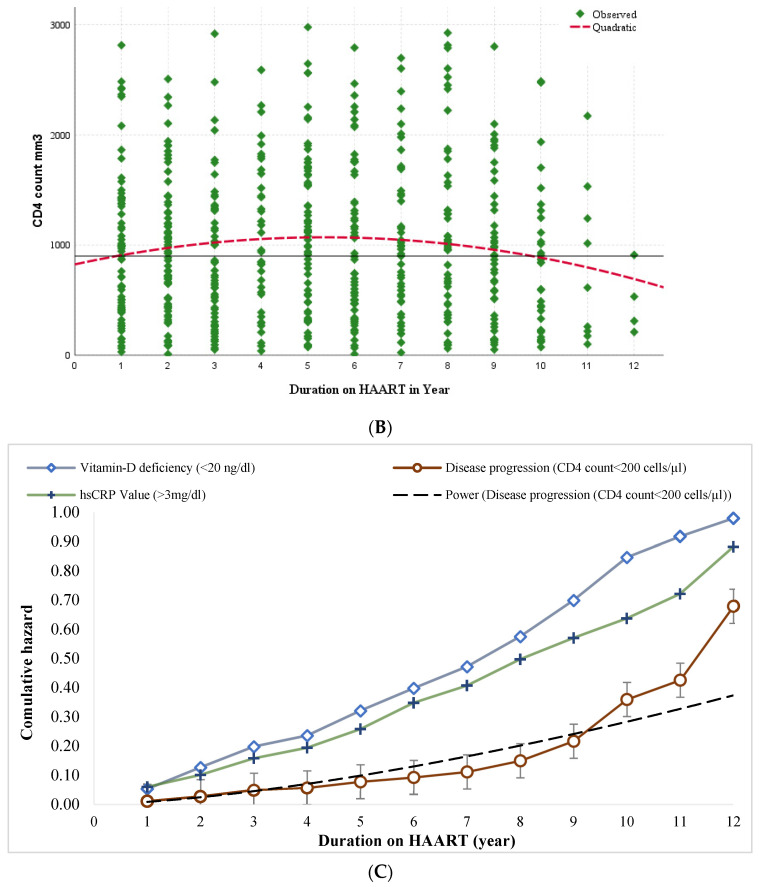
Disease progression (the rate of drop in CD4 count below 200/µL) among children taking HAART in Ethiopia (2007–2019): (**A**) Kaplan–Maier analysis for evaluation of disease progression (**B**) CD4 count distribution over HAART duration (The red dotted lines represent the mean CD4 count and the green dots represent distribution of CD4 count) (**C**) Cumulative hazard on disease progression, vitamin-D deficiency, and hsCRP (>3 mg/dL).

The disease progression rate among children taking HAART was 6.3 per 100 person-years (95% CI = 4.2–8.1). Immunosuppression at the mean duration of 10.5 years was 38.2%, which significantly increased to 67.8% at the 12th year (*p* < 0.01) (Figure 2A,B). Vitamin D at 9 years of follow-up was significantly lower compared to <7 years, *p* = 0.02. The rate of inflammation also showed an increment over time (Figure 2C).

### 3.3. HIV-1 Drug Resistance

HIVDR among children taking HAART in Ethiopia was 14.52% (95% CI = 10.21–12.76), and 6.2% had multi-drug resistance (resistance for more than one drug) mutation. HIV-1 drug resistance was 67.8% among children with a viral load >1000 copies/mL. It was relatively higher among children >10 years old (17.39%). Moreover, those who live in a rural area had 17.54% prevalence of HIVDR compared to the 14.17% among urban dwellers. HIVDR among ambulatory children was 20.0%, while this was 9.97% among bedridden children. About half (48%) of the children taking d4t + 3TC + EFV developed at least one type of drug resistance followed by d4t + 3TC + NVP (16.8%) and AZT + 3TC + NVP (13.6%). About half (50.84%) of the children on HAART for more than 100 months had at least one type of drug resistance mutation. At the regional level, higher rates of at least one drug resistance were found among children in the Afar (27.78%), Tigray (25.64%), and Gambella (19.35%) regions (Supplemental Table S2).

**Figure 3:**
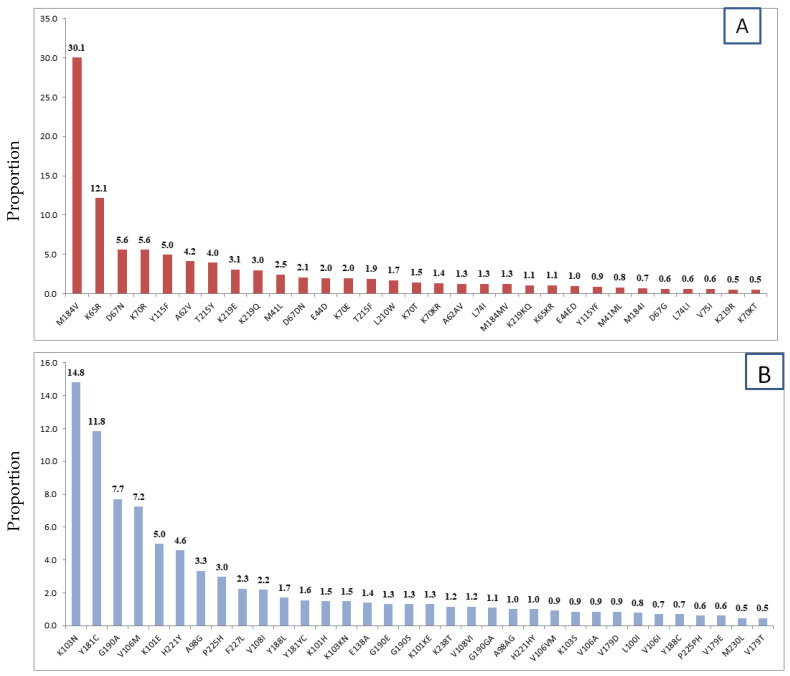
Characteristics of HIVDR mutations among children taking HAART in Ethiopia (2007–2019): (**A**) NRTI associated mutations (**B**) NNRTI associated mutations.

The rate of resistance to NNRTI and NRTI was 11.4% and 10.1%, respectively (Figure 3). The only protease inhibitor (PI) associated with high-level resistance was observed for NFV 1 (0.9%).

High-level resistances were observed for NRTI drugs including 3TC (60.9%), FTC (60.9%), and DDI (43.6%). Resistance levels for ABC, DOR, TDF, and d4T were 34%, 21.8%, 22.7%, and 21.8%, respectively. NNRTIs associated with high levels of drug resistance were NVP (40.0%), EFZ (39.1%), RPV (30.0%), and ETR (12.7%) (Table 2).

Mutations responsible for NRTI resistance were M184V, K65R, D67N, K70R, and Y115F that accounted for 30.1%, 12.1%, 5.6%, 5.6%, and 5.0%, respectively. The common mutations associated with NNRTI resistance were K103N (14.8%), Y181C (11.8%), G190A (7.7%), and V106M (5%) (Figure 3).

### 3.4. Predictors of HIV-1 Disease Progression and Drug Resistance

**Figure 4:**
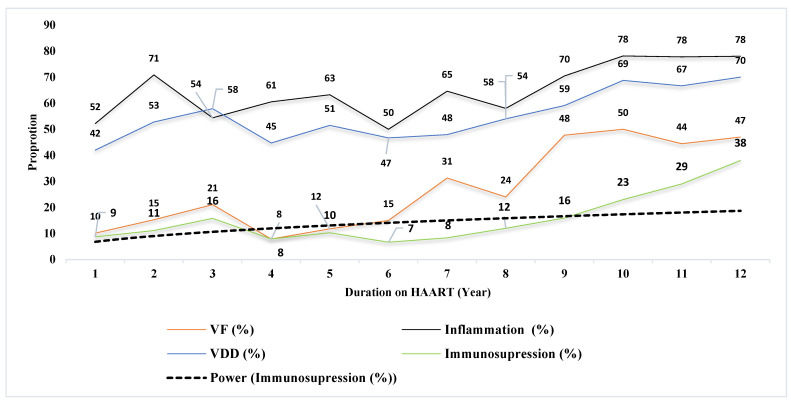
Trends of virologic failure (VL < 1000 copies/mL), immunosuppression (CD4 count < 200 cells/mm^3^), inflammation (hsCRP > 3 mg/dL), and vitamin-D deficiency (<20 ng/dL) among children taking HAART in Ethiopia (2007–2019).

At HAART initiation, virologic failure (VL > 1000 copies/mL), immunosuppression (CD4 count < 200 cells/mm^3^), inflammation (hsCRP > 3 mg/dL), and vitamin-D deficiency (<20 ng/dL) were 10%, 9%, 52%, and 42%, and at 12 years of HAART experience, all significantly increased to 47%, 38%, 78%, and 70%, respectively (Figure 4).

Children who had history of OI [AHR (95% CI = 3.38 (1.84, 6.23)], had HIVDR [AHR (95% CI) = 2.2 (1.4–3.6)], those with VF [AHR (95% CI) = 2.82 (1.21, 3.53)], and vitamin D < 20 ng/mL [AHR (95% CI) = 4.5 (2.1, 9.9)] were associated with an increased hazard of disease progression compared to their respective counterparts (Table 3).

After adjusted analysis, being orphan [AOR (95% CI) = 1.81 (1.2–3.1)], history of drug substitution [AOR (95% CI) = 4.8 (2.1–6.5)], and hemoglobin <12 mg/dL [AOR (95% CI) = 1.24 (1.1–2.0)] had higher odds of developing drug resistance compared to their counterparts (Table 4).

## 4. Discussion

In the current study, the rate of disease progression was 6.3 per 100 person-years, and the prevalence of HIV drug resistance was 14.5% (11.4% for NNRTI and 10.1% for NRTI) among children on first-line antiretroviral therapy in Ethiopia. Immunosuppression at the mean duration of 10.5 years was 38.2%, which nearly doubled (67.8%) at 12 years (*p* < 0.001). Children who had a history of opportunistic infection, vitamin D <20 ng/mL, and drug resistance were significantly associated with a higher hazard of disease progression while being orphaned; drug substitution and hemoglobin <12 mg/dL were independent determinants of drug resistance.

The 6.3 per 100 person-years rate of disease progression among children in the current study was consistent with previous reports [8,10]. Moreover, the experience in children of poor immune recovery at an advanced stage of HAART was also supported by previous studies [4,5,6,7,8,9]. There was a gradual improvement in the CD4 cell count after HAART initiation, which could be indicative of effective HAART response. However, children who were on HAART for more than 10 years showed a rapid disease progression. This was consistent with a finding in India [15] and a similar study among adults in South Africa [16]. This high rate of disease progression after years of exposure to HAART could be explained by a high rate of virologic failure, HAART-associated vitamin-D deficiency, and decreased medication adherence at an advanced stage of the HAART experience [11,12,13,14,15,16,17,18,19,20,21,22,23,24,25]. This finding can also be explained by the fact that a long-time exposure to HAART could lead to drug resistance as a result of selective pressure, which increases viral replication and impacts disease progression [14].

The higher rate of disease progression observed in children with a history of opportunistic infections was consistent with previous findings from South Africa [19] and Kenya [20]. This is because OIs might result from a following decrease in CD4 cell counts [21,22]. Children with an unsuppressed viral load had a higher hazard of disease progression. Unsuppressed viral load was the result of poor medication adherence or treatment failure as a result of HIVDR. In this analysis, adherence was one of the interventions after the first viral load. Hence, unsuppressed viral load is most likely the result of drug resistance, since our analysis also showed drug resistance was a significant predictor of disease progression. On the other hand, vitamin-D deficiency was a significant predictor of disease progression. This can be explained in many ways. Deficiency may lead to disease progression, or malnutrition following disease progression can reciprocally result in vitamin-D deficiency. Longer exposure to HAART could also lead to hypovitaminosis [24,25]. This is also supported by the current findings in Cameroon [26] and another studies [27,28,29,30], which showed a higher decline in vitamin D among children with a low CD4 count and advanced HAART experience. This implies the importance of providing vitamin-D supplement for those with advanced HAART experience and low CD4 count.

The 14.5% prevalence of drug resistance mutation among children on HAART in our study was more than double compared to the 6.7% prevalence of resistance among adults in Ethiopia [31]. This could be explained by the higher rates of medication nonadherence and drug substitution among children. The high prevalence of K103 with NNRTI-associated mutation contradicts with previous reports that showed V106M is the favored NNRTI resistance mutation in HIV-1 infected adults [32]. This could indicate the importance of a population-specific treatment regimen. On the other hand, M184V mutation was also the most common mutation associated with NRTIs. This was consistent with other previous studies conducted in Zimbabwe and Uganda [12,33]. As expected, there were few significant protease inhibitor mutations because of the infrequent use of protease inhibitor containing regimens among the study population. Data from patients in Botswana reported that T215Y occurs in combination with D67N and K70R, rather than with M41L and L210W [34]. Similarly, we identified the presence of M41L in addition to T215Y (with or without L210W). Moreover, K65R is thought to commonly emerge in HIV-1 subtype C [35], which was consistent with our current study.

Our analysis reveals that orphans were almost twice more likely to develop drug resistance, which could result from poor medication adherence, or they might acquire it from their parents who died due to drug resistance. Moreover, drug resistance was higher in children with a history of drug substitution and whose hemoglobin level was <12 mg/dL. This relationship can be bidirectional. Drug substitution during the clinical management of patients might lead to drug resistance [36]. The association between low hemoglobin and drug resistance can be due to advanced disease progression in children with drug resistance [34,37].

This study might have certain limitations. One drawback is that we only studied children who were alive and on treatment, but there might be a significant number lost to follow-up and dead children as a result of poor immunologic outcome. Hence, this study could underestimate disease progression and drug resistance.

## 5. Conclusions

Immunosuppression was increasing over time and drug resistance was also substantially high. This requires that the national HIV program integrate HIVDR testing as part of the individual treatment, particularly for children. Moreover, enhancing routine monitoring of viral load and HIVDR, and providing a vitamin-D supplement during the clinical management, could help improve the immunological outcome. Limiting HAART substitution is also crucial for children taking HAART in Ethiopia.

## Figures and Tables

**Table 1:** Demographic and clinical characteristics of children taking HAART in Ethiopia (2007–2019).

Variable		Frequency	Percent
Gender	Female	273	49.5
	Male	278	50.5
Age (Year)	≤5	76	13.8
	6–10	245	44.5
	>10	230	41.7
Residency	Urban	494	89.7
	Rural	57	10.3
Orphan	Yes	152	27.6
	No	399	72.4
Adherence	Poor	2	0.4
	Fair	10	1.8
	Good	539	97.8
Functional status	Ambulatory	350	89.4
	Bed Ridden	86	11.6
History of HAART substitution	Yes	262	47.5
	No	281	51.0
Viral Load follow-up (copies/mL)	suppressed	433	78.6
	Not suppressed	118	21.4
hsCRP (mg/dL)	≤3	213	38.7
	>3	338	61.3
Vitamin-D (ng/dL)	≤20	284	51.5
	>20	267	48.5
Total		551	100.0

Key: g/dL = gram per deciliter, mg/dL = milligram per deciliter, ng/dL = nanogram per deciliter, hsCRP = highly sensitive C-reactive protein.

**Table 2:** HIV drug resistance among children taking HAART in Ethiopia (2007–2019).

Drug Type	S		PLLR		LLR		IR		HLR	
	Frequency (%)		Frequency (%)		Frequency (%)		Frequency (%)		Frequency (%)	
Darunavir (DRVr)	109	99.1	1.0	0.9	0.0	0.0	0.0	0.0	0.0	0.0
Fos-amprenavir (FPVr)	108	98.2	0.0	0.0	1	0.9	1.0	0.9	0.0	0.0
Indinavir (IDVr)	108	98.2	0.0	0.0	1	0.9	1.0	0.9	0.0	0.0
Loprinavir (LPVr)	108	98.2	0.0	0.0	2	1.8	0.0	0.0	0.0	0.0
Nelfinavir (NFV)	108	98.2	0.0	0.0	1	0.9	0.0	0.0	1.0	0.9
Saquinavir (SQVr)	109	99.1	0.0	0.0	0.0	0.0	1.0	0.9	0.0	0.0
Tipranavir (TPVr)	109	99.1	0.0	0.0	0.0	0.0	1.0	0.9	0.0	0.0
Abacavir (ABC)	27	24.5	19	17.3	19	17.3	11	10.0	34	30.9
Zidovudine (AZT)	80	72.7	11.0	10.0	10	9.1	4.0	3.6	5.0	4.5
Stavudine (D4T)	41	37.3	11.0	10.0	9	8.2	25	22.7	24	21.8
Didanosine (DDI)	26	23.6	16.0	14.5	11	10.0	9.0	8.2	48	43.6
Emtricitabine (FTC)	28	25.5	9.0	8.2	0.0	0.0	6.0	5.5	67	60.9
Lamivudine (3TC)	28	25.5	9.0	8.2	0.0	0.0	6.0	5.5	67	60.9
Tenofovir (TDF)	45	40.9	1.0	0.9	10	9.1	29	26.4	25	22.7
Delavirdine (DOR)	28	25.5	16.0	14.5	8	7.3	34	30.9	24	21.8
Efavirenz (EFV)	14	12.7	31.0	28.2	8	7.3	14	12.7	43	39.1
Etravirine (ETR)	35	31.8	22.0	20.0	10	9.1	29	26.4	14	12.7
Nevirapine (NVP)	14	12.7	31.0	28.2	21	19.1	0.0	0.0	44	40.0
Ritonavir (RPV)	35	31.8	11.0	10.0	18	16.4	13	11.8	33	30.0

Key: S-susceptible; PLLR-potential low level resistance; LLR-low-level resistance; IR-intermediate resistance; HLR-high-level resistance.

**Table 3:** Predictors of disease progression rate among children taking HAART in Ethiopia (2007–2019).

		sig.	CHR (95% CI: Lower, Upper)	sig.	AHR (95% CI: Lower, Upper)
History of OI	Yes	0.00	3.56 (1.90, 6.69)	0.000	3.38 (1.84, 6.23)
	No	Ref.			
Viral Load (Copies/mL)	Suppressed	Ref.			
	Not suppressed	0.00	3.93 (1.90, 4.55)	0.00	2.82 (1.20, 3.53)
HIVDR	Yes	0.03	1.54 (1.24, 3.93)	0.02	2.21 (1.42, 3.64)
	No	Ref.			
Vitamin-D (ng/dL)	≤20	0.00	4.38 (1.97, 9.70)	0.00	4.53 (2.07, 9.94)
	>20	Ref.			

Key: NS-Not significant; Ref.-Reference; CHR-Crude Hazard Ratio; AHR-Adjusted Hazard Ratio; Sig.-Significance level.

**Table 4:** Determinants of HIV drug resistance among children taking HAART in Ethiopia (2007–2019).

Variable		*p*-Value	COR (95% CI)	*p*-Value	AOR (95% CI)
Orphan	Yes	0.08	1.67 (1.18, 2.97)	0.02	1.81 (1.15, 3.08)
	No	Ref.			
History of OI	Yes	0.06	0.42 (0.12, 1.5)	0.19	0.66 (0.36, 1.22)
	No	Ref.			
History of HAART substitution	Yes	0.00	5.499 (2.05, 14.73)	0.04	4.76 (2.09, 6.46)
	No	Ref.			
Hemoglobin (g/dL)	≤12	0.07	2.31 (0.90, 5.90)	0.03	1.24 (1.07, 2.04)
	>12	Ref.			

Key: NS-Not significant; Ref.-Reference; COR-Crude Odds Ratio; AOR-Adjusted Odds Ratio; Sig.-Significance level.

## Data Availability

Since data analysis for other objectives is ongoing, the raw data can be obtained from the first corresponding author.

## Supplementary Materials

The following Supporting Information can be downloaded at: https://www.mdpi.com/article/10.3390/biomedicines11082293/s1, Table S1: Demographic and clinical characteristics of Children taking HAART in Ethiopia (2007–2019); Table S2: Disease progression Immunosupression and HIV drug resistance disaggregated by demographic and clinical characteristics among Children taking HAART in Ethiopia (2007–2019).

## Abbreviations

HAART: Highly Active Antiretroviral Therapy, ART: Active Antiretroviral Therapy, EDTA: Ethylenediaminetetraacetic acid, VL: Viral Load, VF: Virologic Failure, HIVDR: HIV Drug Resistance, OI: Opportunistic Infections, NRTI: Nucleotide Reverse Transcriptase, NNRTI: Non-Nucleotide Reverse Transcriptase, AOR: Adjusted Odds Ratio, hsCRP: Highly sensitive C-Reactive Protein, SSA: Sub-Saharan Africa, PLHIV: People Living with HIV, HF: Health Facility, SNNPR: Southern Nation and Nationalities, DRM: Drug Resistance Mutation. AIDS: Acquired Immune Deficiency Syndrome, CI: Confidence Interval, EPHI: Ethiopian Public Health Institute. HR: Hazard Ratio, Mg/dL: Milligram per Deciliter, ZJU: Zhejiang University.
