# Supplementary material for: HIV-1 Disease Progression and Drug Resistance Mutations among Children on First-Line Antiretroviral Therapy in Ethiopia
Source: Biomedicines. 2023 Aug 18;11(8):2293. doi: 10.3390/biomedicines11082293 (PMC10452141; doi:10.3390/biomedicines11082293)
Supplement: Supplementary file 1 [file biomedicines-11-02293-s001.zip › biomedicines-2485512-SI.pdf]

Table S1. Demographic and clinical characteristics of Children taking HAART in Ethiopia (2007-2019).

Variable		Frequency	Percent
Region	Addis Ababa	97	17.6
	Afar	18	3.3
	Amhara	92	16.7
	Benishangul	25	4.5
	Dire Dawa	4	0.7
	Gambella	31	5.6
	Harari	25	4.5
	Oromia	100	18.1
	SNNPR	66	12.0
	Somali	15	2.7
	Tigray	78	14.2
Interrupted treatment (in the last 1 year)	Yes	26	4.7
	No	525	95.3
Type of HAART	ABC+3TC+EFV	9	1.6
	ABC+3TC+NVP	1	0.2
	AZT+3TC+EFV	77	14.0
	AZT+3TC+NVP	198	35.9
	d4t+3TC+EFV	25	4.5
	d4t+3TC+NVP	167	30.3
	TDF+3TC+EFV	35	6.4
	TDF+3TC+ NVP	2	0.4
Viral Load at baseline (copies/ml)	suppressed	342	62.2
	Not suppressed	208	37.8
Hemoglobin (g/dl)	≤12	273	49.5
	>12	278	50.5
Duration on HAART in Month	1-33	166	30.1
	34-67	142	25.8
	68-100	150	27.2
	101-133	89	16.2
	>133	4	0.7
	**Total**	**551**	**100.0**

Key: abbreviations: g/dl= gram per deciliter, mg/dl= milligram per deciliter, ng/dl=Nano gram per deciliter, hsCRP= highly sensitive C-Reactive Protein

Table S2. Disease progression Immunosupression and HIV drug resistance disaggregated by demographic and clinical characteristics among Children taking HAART in Ethiopia (2007-2019).

Variable		CD4 count (cells/mm^3)				HIV Drug resistance			
		≤ 200	>200	Total	Immunosuppression(%)	No	Yes	Total	HIVDR (%)
Gender	Female	26	247	273	9.52	233	40	273	14.65
	Male	36	242	278	12.95	238	40	278	14.39
Age (Year)	≤ 5	5	71	76	6.58	64	12	76	15.79
	6-10	30	215	245	12.24	217	28	245	11.43
	>10	27	203	230	11.74	190	40	230	17.39
Residency	Urban	54	440	494	10.93	424	70	494	14.17
	Rural	8	49	57	14.04	47	10	57	17.54
HAART exposure prior to Initiation	Yes	0	10	10	0.00	9	1	10	14.00
	No	62	479	541	11.46	462	79	541	10.60
Self-reported adherence	Poor	0	2	2	0.00	1	1	2	50.00
	Fair	2	8	10	20.00	7	3	10	30.00
	Good	60	479	539	11.13	463	76	539	14.10
Functional status at the end of the Follow-up	Ambulatory	26	224	250	10.40	200	50	250	20.00
	Bed Ridden	36	265	301	11.96	271	30	301	9.97
History of OI	Yes	24	82	106	22.64	88	18	106	16.98
	No	38	407	445	8.54	383	62	445	13.93
Type of HAART	ABC+3TC+EFV	1	8	9	11.11	9	0	9	0.00
	ABC+3TC+NVP	0	1	1	0.00	1	0	1	0.00
	AZT+3TC+EFV	12	65	77	15.58	71	6	77	7.79
	AZT+3TC+NVP	26	172	198	13.13	171	27	198	13.64
	d4t+3TC+EFV	3	22	25	12.00	13	12	25	48.00
	d4t+3TC+NVP	15	152	167	8.98	139	28	167	16.77
	TDF+3TC+EFV	2	33	35	5.71	32	3	35	8.57
	TDF+3TC+NVP	0	2	2	0.00	2	0	2	0.00
Viral load (copies/ml)	Suppressed	31	402	433	7.16	433	0	433	0.00
	Not Suppressed	31	87	118	26.27	38	80	118	67.80
Hemoglobin (g/dl)	≤ 12	28	245	273	10.26	238	35	273	12.82
	>12	34	244	278	12.23	233	45	278	16.19
hsCRP (mg/dl)	≤ 3	17	196	213	7.98	198	15	213	7.04
	>3	45	293	338	13.31	273	65	338	19.23
Vitamin-D (ng/dl)	≤ 20	53	231	284	18.66	210	74	284	26.06
	>20	9	258	267	3.37	261	6	267	2.25
Duration on HAART (Month)	<33					151	15	166	9.04
	34-67					128	14	142	9.86
	68-100					123	27	150	18.00
	101-133					66	23	89	25.84
	>133					3	1	4	25.00
Total (%)					11.25				14.52
